# Numerical Simulations of Single-Step Holographic Interferometry for Split-Ring Metamaterial Fabrication

**DOI:** 10.3390/nano15020086

**Published:** 2025-01-08

**Authors:** Zhiming Qi, Wenyao Liang

**Affiliations:** 1 Guangdong Open University, Guangdong Polytechnic Institute, Guangzhou 510091, China; zmqi@gdrtvu.edu.cn; 2 School of Physics and Optoelectronics, South China University of Technology, Guangzhou 510640, China

**Keywords:** metamaterials, single-step holographic interferometry, beam configuration, polarization combinations, numerical simulations

## Abstract

Artificial microstructures, especially metamaterials, have garnered increasing attention in numerous applications due to their rich and distinctive properties. Starting from the principle of multi-beam interference, we have theoretically devised a beam configuration consisting of six symmetrically distributed coherent beams to generate two-dimensional microstructures with diverse shapes of unitcells under different polarization combinations. In particular, a split-ring metamaterial template is achieved with two adjacent circularly and four linearly polarized beams with such single-step holographic interferometry. Furthermore, simulation results show that the orientation and shape of the split-ring unitcell can be accurately adjusted by controlling the polarization position, polarization degree, or power ratio of the coherent beams. The optimal parameters to produce a high-quality split-ring metamaterial with a contrast higher than 0.97 are obtained. These results provide useful guidance for the effective and low-cost fabrication of metamaterials with diverse unitcells.

## 1. Introduction

Nowadays, artificial microstructures (especially metamaterials) have been attracting continuously growing attention because of their unique or extreme thermal, structural, mechanical, and dynamic properties, which are highly demanded in fundamental research and numerous applications [1,2,3]. Metamaterials refer to composite materials with artificially designed structures to exhibit extraordinary properties that natural materials do not possess, including metal or dielectric periodic structures with their sizes much smaller than the working wavelength. The unique properties of metamaterials come from artificially designed special structures, rather than the constituent materials. Metamaterials can be described by the equivalent dielectric function *ε* and equivalent magnetic permeability *μ*. J.B. Pendry classified all materials according to their symbols [4], including right-handed materials (*ε* > 0, *μ* > 0) (such as common natural materials), left-handed materials (*ε* < 0, *μ* < 0), and single negative materials (*ε* > 0, *μ* < 0 or *ε* < 0, *μ* > 0). Among them, metamaterials mainly refer to left-handed materials [5,6,7] and single negative materials [8,9]. Metamaterials have strong manipulation abilities on physical fields, which can break through the physical limits of traditional materials, and possess a series of interesting effects such as negative refraction [10], super-resolution imaging [11], perfect absorption [12,13], and optical invisibility [14]. They have great potential in various fields such as communication, radar, microelectronics, and medical imaging.

In the fabrications of metamaterials, various methods have been proposed, including a kirigami-inspired method [15], electron beam etching [16,17], 3D printing [18], self-assembly [19], and laser holographic interferometry [20]. Among them, the kirigami-inspired method utilizes a focused ion beam (FIB) technique to accurately cut out two-dimensional nano-pattern in a film and then uses the stress effect generated by the same FIB technique to “pull” the nano-pattern into a complex three-dimensional shape to form a metamaterial. This method requires high-precision FIB equipment and involves a complex operational process. The electron beam etching method employs high-energy electron beams to strike the surface of materials, triggering physical and chemical reactions to obtain micro-scale metamaterial structures. The etching process is relatively slow and prone to causing material damage. The working principle of 3D printing is to slice a three-dimensional model into two-dimensional layers, and then stack materials (such as plastics, metals, or resins) layer by layer on a platform to form metamaterial structures with specific physical properties. It is associated with relatively high costs, slow printing speeds, and potential limitations in terms of printer precision and material selection. The self-assembly method leverages the intermolecular or inter-nanoparticle forces to spontaneously organize into ordered metamaterial structures. It can only produce certain types of structures, and it is challenging to precisely control the morphology of the final structure.

Different from the previous method, laser holographic interferometry utilizes the interference of multiple coherent beams to generate periodic patterns and records them on appropriate materials to obtain the desired microstructure. It has unique advantages such as large area, low cost, high efficiency and flexibility, and rich interference patterns simultaneously. Laser holographic interferometry has been used to produce various microstructures such as two- and three-dimensional periodic structures and quasicrystalline structures [21,22,23,24,25,26] and can also be used for multiple exposures to introduce defects according to practical needs. It is worth noting that laser holographic interferometry is a highly promising technology for generating one-, two-, and three-dimensional microstructures with various unitcell shapes including photonic crystals and metamaterials with only one-step exposure.

In this work, we start from the principle of multi-beam interference and theoretically study the implementation mechanism and beam configuration for two-dimensional split-ring metamaterial with one-step holographic interferometry via numerical simulations. The influence factors such as polarization position, polarization degree, and power ratio of the coherent beams on the split-ring unitcell of the metamaterial are systematically studied, and the optimal parameters of beam configuration are obtained finally. These results provide useful references for the development of high-efficiency and low-cost metamaterial fabrication, which also indicates the advantages of laser holographic interferometry in fabricating periodic microstructures with a special shape of unitcell.

## 2. Methods

### 2.1. Principle of Multi-Beam Holographic Interferometry

**Figure 1:**
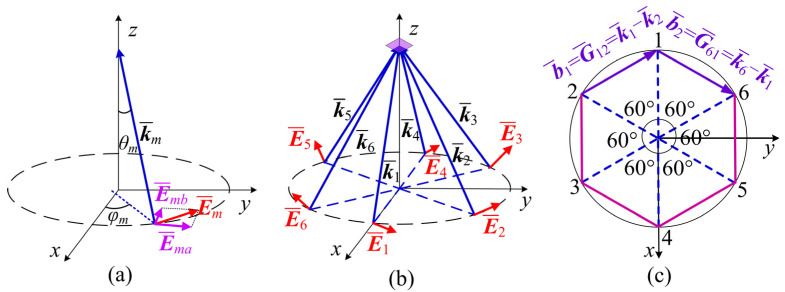
(**a**) Polarization decomposition of the *m*th light beam. (**b**) Beam configuration of six light beams. (**c**) The projected beam configuration and reciprocal basis vectors.

The principle of multi-beam interference is the physical basis of holographic interferometry. Due to the excellent coherence of lasers, they are often selected as light sources in laser holography experiments and can be approximated by plane light waves. We assume that there are *N* plane light waves with the same frequency at a wavelength of *λ* = 445 nm (*m* = 1, 2, …, *N*) involved in interference, as shown in Figure 1a. The *m*th beam is expressed as [27]

(1) $${\bar{E}}_{m}(\bar{r})={\bar{E}}_{m}\exp[i({\bar{k}}_{m}\cdot \bar{r}+\delta_{m}),$$

where ${\bar{E}}_{m}$, ${\bar{k}}_{m}$, and $\delta_{m}$ are the complex amplitude vector, wave vector, and initial phase of the *m*th plane light beam, respectively. Due to the fact that elliptically polarized light can degrade into linearly or circularly polarized light under specific conditions and in order to facilitate the analysis of the influence of polarization, the electric vector of each beam is described in the form of elliptically polarized light. Specifically, the polarization of ${\bar{E}}_{m}$ is decomposed into two perpendicular vibration components ${\bar{E}}_{ma}$ and ${\bar{E}}_{mb}$ (the major and minor axes of the ellipse, respectively) with an initial phase difference of π/2. Therefore, the *m*th plane light beam can be described as follows [24]:

(2) $${\bar{E}}_{m}(\bar{r})=E_{ma}e^{i({\bar{k}}_{m}\cdot \bar{r}+\delta_{m})}{\bar{e}}_{ma}+E_{mb}e^{i({\bar{k}}_{m}\cdot \bar{r}+\delta_{m}-\frac{\pi }{2})}{\bar{e}}_{mb},$$

where ${\bar{e}}_{ma}$ and ${\bar{e}}_{mb}$ are the unit vectors of ${\bar{E}}_{ma}$ and ${\bar{E}}_{mb}$, respectively. According to the principle of the superposition of light waves, when *N* monochromatic plane waves with the same amplitude and initial phase are coherent with each other, the spatial distribution of their interference light field is expressed as follows [24]:

(3) $$\begin{array}{c} I(\bar{r})={\bar{E}}_{total}(\bar{r})\cdot {\bar{E}}_{total}^{*}(\bar{r})={\left|\sum_{m}E_{ma}\exp[i({\bar{k}}_{m}\cdot \bar{r}+\delta_{m})]{\bar{e}}_{ma}+\sum_{m}E_{mb}\exp[i({\bar{k}}_{m}\cdot \bar{r}+\delta_{m}-\pi /2)]{\bar{e}}_{mb}\right|}^{2}\\ =\sum_{m}\left(E_{ma}^{2}+E_{mb}^{2}\right)+\sum_{m<n}2E_{ma}E_{na}{\bar{e}}_{ma}\cdot {\bar{e}}_{na}\cos({\bar{G}}_{mn}\cdot \bar{r}+\delta_{mn})+\sum_{m<n}2E_{mb}E_{nb}{\bar{e}}_{mb}\cdot {\bar{e}}_{nb}\cos({\bar{G}}_{mn}\cdot \bar{r}+\delta_{mn})\\ +\sum_{m<n}2E_{ma}E_{nb}{\bar{e}}_{ma}\cdot {\bar{e}}_{nb}\cos({\bar{G}}_{mn}\cdot \bar{r}+\delta_{mn}-\pi /2)+\sum_{m<n}2E_{mb}E_{na}{\bar{e}}_{mb}\cdot {\bar{e}}_{na}\cos({\bar{G}}_{mn}\cdot \bar{r}+\delta_{mn}+\pi /2) \end{array}$$

where ${\bar{G}}_{mn}={\bar{k}}_{m}-{\bar{k}}_{n}$ is the wave vector difference, and $\delta_{mn}=\delta_{m}-\delta_{n}$ is the initial phase difference. From Equation (3), one can find that (i) multi-beam interference produces a periodic microstructure (such as a photonic crystal or metamaterial), and the type of the microstructure only depends on ${\bar{G}}_{mn}$. Arbitrary double beams that are not aligned in a line can interfere with each other to produce a one-dimensional stripe, and the stacking of all one-dimensional stripes with various orientations form a two- or three-dimensional periodic microstructure. (ii) The relative strengths of various one-dimensional stripes depend on the amplitude and polarization of each light beam (such as $E_{ma}E_{na}{\bar{e}}_{ma}\cdot {\bar{e}}_{na}$), and their superposition determines the shape of the unitcell, finally. In other words, the shape of the unitcell and the contrast of the microstructure depend on the intensities and polarization combinations of the *N* coherent light beams sensitively.

### 2.2. Formation Mechanism of Periodic Microstructures

By combining the principle of multi-beam interferometry and the type of microstructure to be fabricated, we can determine the required beam configuration, which includes the number of beams and the spatial distributions of all beams. The basic design idea is as follows: (i) Starting from the basis vector ${\bar{a}}_{l}$ (*l* = 1, 2, 3) of a desired microstructure, we use the transformation relationship between the real and reciprocal spaces to determine the reciprocal basis vector ${\bar{b}}_{l}$(*l* = 1, 2, 3); (ii) we then choose three ${\bar{G}}_{mn}$ to correspond with ${\bar{b}}_{l}$(*l* = 1, 2, 3) one by one as the reciprocal basis vectors for generating the desired microstructure. It should be noted that for a periodic lattice, those not chosen ${\bar{G}}_{mn}$ will not destroy the translational symmetry of the structure; (iii) we further calculate the propagation direction ${\bar{k}}_{m}$ of each beam and provide the detailed beam parameters of all involved beams.

Now, we discuss the formation mechanism of one-, two-, and three-dimensional microstructures using laser holographic interferometry. Based on the above analyses and Equation (3), when there are two light beams (i.e., *N* = 2), only one wave vector difference ${\bar{G}}_{12}={\bar{k}}_{1}-{\bar{k}}_{2}$ is produced, leading to a one-dimensional interference stripe (i.e., a one-dimensional microstructure). When *N* = 3, three beams can produce three wave vector differences of ${\bar{G}}_{12}={\bar{k}}_{1}-{\bar{k}}_{2}$, ${\bar{G}}_{23}={\bar{k}}_{2}-{\bar{k}}_{3}$, and ${\bar{G}}_{13}={\bar{k}}_{1}-{\bar{k}}_{3}$. However, there exist only two independent wave vector differences such as ${\bar{G}}_{12}$ and ${\bar{G}}_{23}$ because of ${\bar{G}}_{13}={\bar{G}}_{12}+{\bar{G}}_{23}$. When *N* > 3, there will be more than three independent wave vector differences, which can be divided into two cases. When all wave vector differences ${\bar{G}}_{mn}$ locate within the same plane, they interfere with each other to generate a two-dimensional complex lattice; otherwise, their interference results in a three-dimensional microstructure such as a woodpile structure [28]. In other words, a microstructure with a special shape of unitcell can be formed by one-step holographic interferometry without multiple exposures, which is very convenient for practical applications such as fabricating metamaterials.

## 3. Results

### 3.1. Realization of Two-Dimensional Annular Microstructure

In the following text, we will discuss how to generate a two-dimensional annular microstructure, which is important for further generating a split-ring metamaterial. The beam configuration is shown in Figure 1b. There are six coherent light beams symmetrically distributed around the *z*-axis, intersecting at a zone on the *z*-axis. Their projections in the *xoy* plane are shown in Figure 1c. For convenience, the propagating direction of *k_m_* is described by the polar angle *θ_m_* (defined as the angle between the *m*th beam and the *z*-axis) and the azimuth angle *φ_m_* (defined as the angle between its projection in the *xoy* plane and the *x*-axis). In terms of polarization decomposition, the direction of the long axis ${\bar{e}}_{ma}$ is defined as the intersection of the plane perpendicular to the wave vector *k_m_* and the *xoy* plane, while the direction of the short axis ${\bar{e}}_{mb}$ can be obtained by cross multiplication of ${\bar{k}}_{m}\times {\bar{e}}_{ma}$. Such six coherent light beams can interfere with each other to produce a microstructure with a special unitcell. The clarity of the interference pattern is an important indicator which is described by interference contrast *V* [24]:

(4) $$V=\frac{I_{max}-I_{\min}}{I_{max}+I_{\min}}$$

where *I_max_* and *I_min_* are the maximum and minimum values of the interference light intensities, respectively. When *I_min_* = 0, *V* is equal to 1, and the interference strips are the clearest, which is called complete coherence; when *I_max_* = *I_min_*, *V* is equal to zero and the light intensity appears completely bright or completely dark with no interference stripes, which is called completely incoherent; when 0 < *I_min_* < *I_max_* and thus 0 < *V* < 1, the interference stripes can be observed but not the clearest, which is called partially coherent. The higher the contrast *V*, the more advantageous it is for conducting interference experiments.

In order to obtain an intuitive understanding of the formation of microstructures, we use the Matlab tool to write and debug a program based on Formulas (1)–(4) to simulate the interference behaviors of multi-beam holographic interferometry. The main steps of our program are described as follows: (i) According to the lattice type to be fabricated, we set all necessary parameters of beam configuration, including the number of coherent light beams (*N*), the wave vector direction of *k_m_* (*m* = 1, …, *N*) described by the polar angle *θ_m_* and the azimuth angle *φ_m_*, the polarization components *E_ma_* and *E_mb_* for each beam, and their initial phases. (ii) Next, we use the Matlab tool, which contains rich functions and matrix operations to write and debug an effective program to calculate the spatial intensity distribution of the interference of *N* coherent beams. After the calculation area and grid size are given, together with all the parameters given in step (i), the intensity of *I*(*x*, *y*, *z*) at each grid point is calculated. The contrast *V* is also given with Formula (4). (iii) Finally, we plot out the figures to show the intensity distributions under various conditions of polarization combinations and power ratios of the *N* coherent beams. We have proved that our program works well by comparing the simulation results carried out by our program and those in the reported literature under the same beam parameters.

Without loss of generality, in the following calculations, we assume that the six beams have the same intensities, and their initial phases are zero. For the convenience of characterizing the polarization of each beam, the polarization degree parameter is defined as *γ_m_* = *E_mb_*/*E_ma_* (*m* = 1, 2,…, 6), which is the ratio of *E_mb_* and *E_ma_* components of the *m*th beam. Several special polarizations are explained as follows: when *γ_m_* = 0 (meaning *E_mb_* = 0), it represents a linearly polarized light vibrating along ${\bar{e}}_{ma}$; when *γ_m_* = 1 (meaning *E_mb_* = *E_ma_*), it represents a circularly polarized light; when *γ_m_* >> 1, e.g., *γ_m_* = 10^6^ (i.e., *E_mb_* >> *E_ma_*), it can be regarded as a linearly polarized light vibrating along the direction ${\bar{e}}_{mb}$. In other cases, the light beams are elliptical polarized light beams.

**Figure 2:**
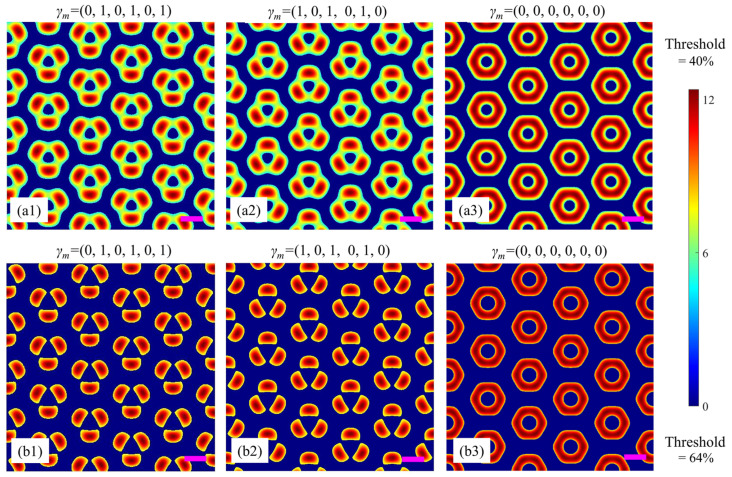
Simulation results for different polarization combinations. (**a1**–**a3**) and (**b1**–**b3**) are obtained under relative intensity thresholds of 40% and 64%, respectively. Purple scale bars: equal to the interference wavelength *λ*.

The actual fabrication procedure for split-ring metamaterials is briefly described as follows: firstly, a template with an array of split rings is obtained on a photoresist using the proposed multi-beam holography method here together with a series of operations such as post-drying and development, then, a metal material (e.g., gold) is evaporated onto the template, and after lifting off the template, the actual split-ring metamaterial is obtained. The current work focuses on the first step to generating metamaterial templates, which is crucial for the actual fabrication of metamaterials. The split-ring array can be recorded with a positive photoresist (e.g., diazonaphthoquinone (DNQ)-based photoresist) under an appropriate intensity threshold. Figure 2(a1) is a simulation example based on the beam configuration in Figure 1 and the corresponding parameters in Table 1. Among them, beams 1, 3, and 5 are linearly polarized along ${\bar{e}}_{ma}$, while beams 2, 4, and 6 are circularly polarized lights. In other words, the polarization degrees (*γ_m_* = *E_mb_*/*E_ma_*) of the six beams are (0, 1, 0, 1, 0, 1). For these parameters, the interference result is a microstructure composed of three sets of nested simple triangular lattices. The unitcell of such a microstructure contains three ‘atoms’ distributed in an equilateral triangle. Similarly, when the polarizations of beams 1, 3, 5, and 2, 4, 6 are exchanged, i.e., the polarization degrees (*γ_m_* = *E_mb_*/*E_ma_*) of the six beams become (1, 0, 1, 0, 1, 0), another similar microstructure but with its unitcell rotated 180° is formed, as shown in Figure 2(a2). Furthermore, when all of the six beams are linearly polarized with their polarization degrees to be (0, 0, 0, 0, 0, 0), they interfere with each other to form an interesting microstructure with an annular unitcell, as shown in Figure 2(a3). The lattice constant (i.e., the distance between the centers of the two nearest annular unitcells) is calculated to be 2.3*λ* ≈ 1 μm. For comparison, the interference wavelength *λ* = 445 nm is denoted by the purple scale bars in all the simulation figures in the following discussions.

It should be noted that in practical holographic experiments, the relative intensity threshold is also an important parameter to influence the unitcell. By combining with the characteristics of the photoresist, the “threshold” can be easily controlled through the exposure time to achieve a desired metamaterial template in practical fabrication. For comparison, Figure 2(a1–a3) are obtained under a relative intensity threshold of 40%, while Figure 2(b1–b3) are the simulation results under a higher relative intensity threshold of 64%. Obviously, it is convenient to adjust the threshold and polarization combination of the coherent light beams to achieve different microstructures with desired unitcells.

### 3.2. Realization of Split-Ring Metamaterial

**Figure 3:**
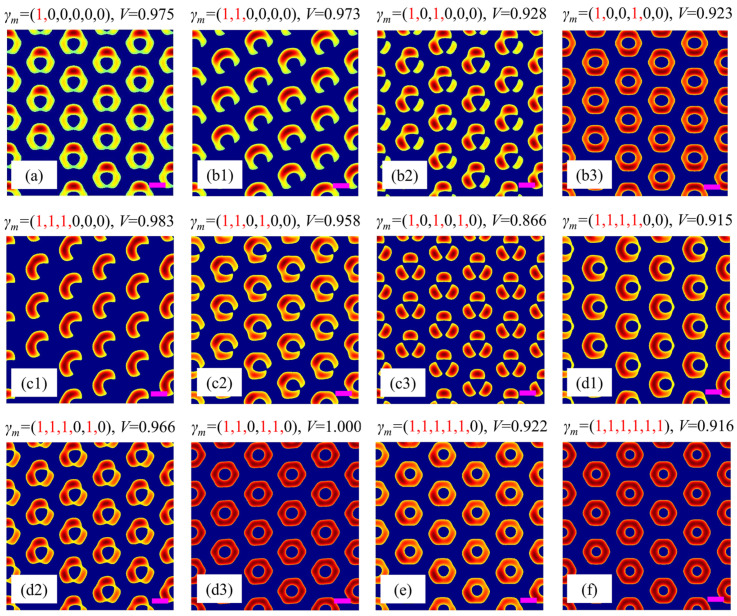
Simulation results for different polarization combinations: (**a**) one, (**b1**–**b3**) two, (**c1**–**c3**) three, (**d1**–**d3**) four, (**e**) five, or (**f**) six beams are circularly polarized (*γ_m_* = 1), while the left beams are kept as linearly polarized (*γ_m_* = 0). The red number 1 and black number 0 in *γ_m_* represent the circularly and linearly polarized beams, respectively. Purple scale bars: equal to the interference wavelength *λ*.

From the above simulation example of annular microstructures, one can find that the polarization combination plays an important role in the formation of special shapes of unitcells in multi-beam holographic interferometry. Here, we proceed to study the evolution of the unitcell under different polarization combinations to generate a split-ring metamaterial. Considering different types of polarization (such as linear, elliptical, or circular polarization) and various polarization degrees, there are a number of polarization combinations for the six coherent beams, which makes it difficult to analyze all cases of polarization combinations. Inspired by Figure 2, we find that circular polarization is a key point in producing an annular microstructure. Therefore, for simplicity, we only consider combinations of circular and linear polarizations for the six beams to analyze the optimal polarization combination to produce a split-ring metamaterial. Figure 3 shows the simulation results of different polarization combinations, i.e., one, two, three, four, five, or six beams are circularly polarized (*γ_m_* = 1), while the left beams are kept as linearly polarized (*γ_m_* = 0). All simulation results are obtained under a relative intensity threshold of 64%.

For the case of only one circularly polarized beam among the six beams, we only need to consider one typical polarization combination of *γ_m_* = (1, 0, 0, 0, 0, 0) due to the six-fold rotational symmetry of the beam configuration shown in Figure 1b. Figure 3a shows that when beam 1 is changed from linear to circular polarization, the intensity distribution of the unitcell deviates from the perfect annular shape in Figure 2(b3) for *γ_m_* = (0, 0, 0, 0, 0, 0), leading to a small gap at the bottom of the unitcell and a contrast of *V* = 0.975. This is because the circularly polarized beam has a less effective component contributing to the interference with other linear beams. It is worth noting that this type of unitcell has a similar shape to the split-ring metamaterial, which means that multi-beam holographic interferometry combined with beam polarization control can be used to fabricate split-ring metamaterial template as discussed in Section 3.1, and then the practical metamaterial can be produced by reversely filling with metal material with evaporation and subsequent lifting off operation, providing an effective approach for the fabrication of metamaterials.

When the number of circularly polarized beams increases to two, there exist three sub-cases due the six-fold rotational symmetry of the beam configuration, i.e., adjacent case [*γ_m_* = (1, 1, 0, 0, 0, 0)], separated case [*γ_m_* = (1, 0, 1, 0, 0, 0)], and opposite case [*γ_m_* = 1, 0, 0, 1, 0, 0)]. The simulation results are shown in Figure 3(b1–b3). It is seen that the shapes of the unitcells are very different from each other. For the adjacent case in Figure 3(b1), the unitcell is also a split ring (*V* = 0.973) but with a much better intensity distribution than that shown in Figure 3a. The gap of the split ring is along the bottom-right direction. For the separated case in Figure 3(b2), the unitcell consists of two connected ‘atoms’ and a separated long-shaped ‘atom’ (*V* = 0.928), while for the opposite case in Figure 3(b3), the unitcell is still annular but with lower intensity on its left and right parts (*V* = 0.923). These differences can be understood by the symmetry of the polarization combinations of the six beams. A circularly polarized beam has only a half-effective component to interfere with the other beams. When the circularly polarized beams are located at different positions, various intensity distributions appear for different polarization combinations.

Similarly, when the number of circularly polarized beams increases to three or four, there also exist three sub-cases, respectively. As for the case of three circularly polarized beams, their polarization combinations are *γ_m_* = (1, 1, 1, 0, 0, 0), (1, 1, 0, 1, 0, 0), and (1, 0, 1, 0, 1, 0). The simulation results are shown in Figure 3(c1–c3). Their unitcells are crescent-shaped (*V* = 0.983), small gap split ring (*V* = 0.958), and three-separate-spot (*V* = 0.866), respectively. As for the case of the four circularly polarized beams, their polarization combinations are *γ_m_* = (1, 1, 1, 1, 0, 0), (1, 1, 1, 0, 1, 0), and (1, 1, 0, 1, 1, 0), respectively. The results in Figure 3(d1–d3) indicate that their unitcells are all annular but with different degrees of distortion, and their contrasts are *V* = 0.916, 0.966, and 1, respectively.

When the number of circularly polarized beams further increases to five or six, there exists only one set of polarization combination, respectively, i.e., *γ_m_* = (1, 1, 1, 1, 1, 0) or (1, 1, 1, 1, 1, 1). For the case of (1, 1, 1, 1, 1, 0), the unitcell is annular but with a weak intensity at its upper-right part (*V* = 0.922), as shown in Figure 3e. While for the case of (1, 1, 1, 1, 1, 1), since the polarizations and the propagation directions of all beams are six-fold symmetrical with respect to the *z*-axis, the unitcell becomes a perfect annular shape again but with a relative lower contrast of *V* = 0.916, as shown in Figure 3f, when compared with the annular unitcell (*V* = 1) shown in Figure 2(b3) for *γ_m_* = (0, 0, 0, 0, 0, 0).

Based on the above analyses, one can find that the optimal polarization combination to create a split-ring metamaterial is the adjacent case of *γ_m_* = (1, 1, 0, 0, 0, 0). Therefore, in the following text, we will start from this set of configuration parameters to further discuss the influence of other important factors on the unitcell.

### 3.3. Influence of Polarization Position, Polarization Degree, and Power Ratio

**Figure 4:**
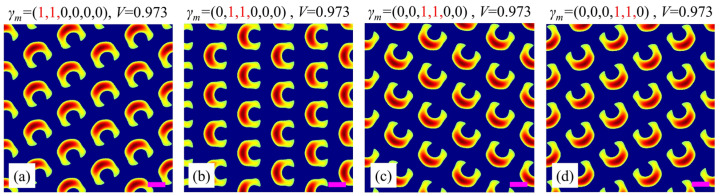
Simulation results for different polarization positions of adjacent circularly polarized beams. (**a**) (1, 2) beams; (**b**) (2, 3) beams; (**c**) (3, 4) beams; (**d**) (4, 5) beams. The red number 1 and black number 0 in *γ_m_* represent the circularly and linearly polarized beams, respectively. Purple scale bars: equal to the interference wavelength *λ*.

Firstly, we investigate the influence of the position of the adjacent circularly polarized beams on the split-ring metamaterial. As shown in Figure 4, when *γ_m_* = (1, 1, 0, 0, 0, 0), the gap of the split ring points along the bottom-right direction with an angle of −60° with respect to the horizontal direction. When *γ_m_* = (0, 1, 1, 0, 0, 0), the gap of the split ring rotates at an angle of 60° counterclockwise, pointing along the right direction. Similarly, when *γ_m_* = (0, 0, 1, 1, 0, 0) and (0, 0, 0, 1, 1, 0), the gap of the split ring continues to rotate 60° counterclockwise, as shown in Figure 4c,d. These results imply that one can achieve a split-ring metamaterial with a specific gap direction by simply changing the positions of the adjacent circularly polarized beams, which is convenient and beneficial for the practical fabrication of split-ring metamaterials.

**Figure 5:**
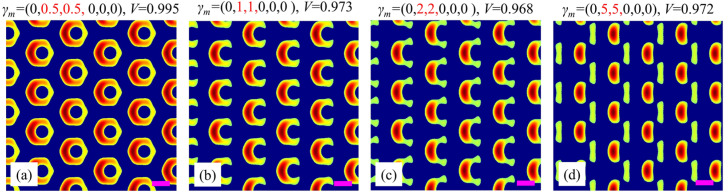
Simulation results for different polarization degrees of adjacent beams 2 and 3: (**a**) (0.5, 0.5); (**b**) (1, 1); (**c**) (2, 2); (**d**) (5, 5). The red numbers in *γ_m_* indicate the polarization degrees of beams 2 and 3; while the black number 0 in *γ_m_* represents that beams 1, 4, 5, 6 are linearly polarized beams. Purple scale bars: equal to the interference wavelength *λ*.

Next, we discuss the influence of the polarization degree of the adjacent beams on the split-ring metamaterial. Without loss of generality, the polarization of beams 2 and 3 are changed simultaneously, while all the left beams are kept unchanged as linear polarized beams. Figure 5a–d present the simulation results as the polarization degree increases. When *γ_m_* is changed from (0, 0.5, 0.5, 0, 0, 0) to (0, 1, 1, 0, 0, 0) (i.e., beams 2 and 3 vary from elliptical to circular polarization simultaneously), the unitcell changes from a slightly asymmetrical annular shape to a split-ring shape, as shown in Figure 5a,b. When the polarization degrees of beams 2 and 3 further increase to 2 (i.e., to be elliptical polarization but with its major axis along ${\bar{e}}_{mb}$), it is still a split-ring unitcell but with its two arms being much weaker, as shown in Figure 5c. Moreover, when their polarization degrees further increase to 5 (i.e., to be elliptical polarization with higher eccentricity), the unitcell degenerates from a split ring to a complex one consisting of a strong short-shaped ‘atom’ and a weak long-shaped ‘atom’, as shown in Figure 5d. These results demonstrate the sensitive dependence of the unitcell on the polarization degree. This property makes it possible to accurately adjust the arm shape of the split-ring unitcell within a certain range by simply changing the polarization degree of the adjacent beams.

**Figure 6:**
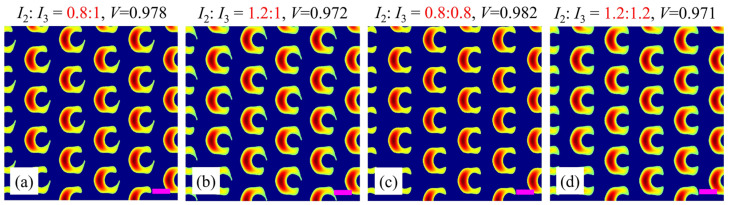
Simulation results for different power ratios of beams 2 to 3: (**a**) 0.8:1; (**b**) 1.2:1; (**c**) 0.8:0.8; (**d**) 1.2:1.2. The red numbers indicate the power ratios of beams 2 and 3. Purple scale bars: equal to the interference wavelength *λ*.

Finally, we study the influence of the power ratio of circularly polarized beams 2 and 3 (*I*_2_:*I*_3_) on the split-ring metamaterial. For convenience, the powers of the other beams are kept unchanged as *I*_1_:*I*_4_:*I*_5_:*I*_6_ = 1:1:1:1. The simulation results are shown in Figure 6. For *I*_2_:*I*_3_ = 0.8:1, the lower arm of the split-ring unitcell is longer than the upper one, which is a reversal of the case of *I*_2_:*I*_3_ = 1.2:1, as shown in Figure 6a,b. Furthermore, when both powers of beams 2 and 3 vary simultaneously, i.e., the power ratio of *I*_2_:*I*_3_ increases from 0.8:0.8 to 1.2:1.2, both arms become thicker and thicker simultaneously. In addition, it is noted that the values of contrast *V* for all these cases stay at a stable level around 0.975. Undoubtedly, these results provide an efficient way to control each arm or both arms of the split-ring metamaterial by changing the power ratio of beams 2 and 3 independently or simultaneously. As a result, based on the aforementioned discussions, one can obtain the optimal beam configuration to generate a high-quality split-ring metamaterial (*V* > 0.97) in the adjacent case with *γ_m_* = (0, 1, 1, 0, 0, 0), which generates the eventually produced split-ring metamaterial template as shown in Figure 5b. Its lattice constant is calculated to be 2.3*λ* ≈ 1 μm, and such a structure typically operates at near-infrared spectrum. Of course, the parameters can be further adjusted to generate other desired microstructures.

## 4. Conclusions

In conclusion, this work theoretically studies the implementation mechanism and beam configuration to generate microstructure by one-step laser holographic interferometry according to the principle of multi-beam interference. By using the beam configuration of six symmetrical coherent beams, abundant microstructures with various unitcell shapes are obtained under different polarization combinations. In particular, a split-ring metamaterial is generated with two adjacent circularly polarized beams and four linearly polarized beams. Moreover, the influence of polarization position, polarization degree, and power ratio of the beams on the split-ring unitcell of the metamaterial are systematically studied, and the optimal parameters of beam configuration to form split-ring metamaterial with high-intensity contrast (*V* > 0.97) are finally given. The above research not only enriches the connotation of laser holographic interferometry but also provides useful guidance for the effective and low-cost fabrication of metamaterials.

## Figures and Tables

**Table 1:** Beam configuration parameters for Figure 2(a1) where *γ_m_* = (0, 1, 0, 1, 0, 1).

${\bar{k}}_{m}$	$\mathrm{Direction}\;\mathrm{of}\;\bar{k}$		${\bar{E}}_{ma}$			${\bar{E}}_{mb}$		
	*θ_m_* (°)	*φ_m_* (°)	$\left|{\bar{E}}_{ma}\right|$	*θ_ma_* (°)	*φ_ma_* (°)	$\left|{\bar{E}}_{mb}\right|$	*θ_mb_* (°)	*φ_mb_* (°)
${\bar{k}}_{1}$	30	180	1	90	270	0	60	0
${\bar{k}}_{2}$	30	240	$\frac{\sqrt{2}}{2}$	90	330	$\frac{\sqrt{2}}{2}$	60	60
${\bar{k}}_{3}$	30	300	1	90	30	0	60	120
${\bar{k}}_{4}$	30	0	$\frac{\sqrt{2}}{2}$	90	90	$\frac{\sqrt{2}}{2}$	60	180
${\bar{k}}_{5}$	30	60	1	90	150	0	60	240
${\bar{k}}_{6}$	30	120	$\frac{\sqrt{2}}{2}$	90	210	$\frac{\sqrt{2}}{2}$	60	300

## Data Availability

The original contributions presented in this study are included in the article. Further inquiries can be directed to the corresponding author.
